# Assessing police topological efficiency in a major sting operation on the dark web

**DOI:** 10.1038/s41598-019-56704-4

**Published:** 2020-01-09

**Authors:** Bruno Requião da Cunha, Pádraig MacCarron, Jean Fernando Passold, Luiz Walmocyr dos Santos, Kleber A. Oliveira, James P. Gleeson

**Affiliations:** 1 Rio Grande do Sul Superintendency, Federal Police, Porto Alegre, 90160-093 Brazil; 2 MACSI, Department of Mathematics and Statistics, University of Limerick, Limerick, V94 T9PX Ireland; 3 Centre for Social Issues Research, University of Limerick, Limerick, V94 T9PX Ireland

**Keywords:** Applied mathematics, Complex networks

## Abstract

The networked nature of criminals using the dark web is poorly understood and infrequently studied, mostly due to a lack of data. Rarer still are studies on the topological effectiveness of police interventions. Between 2014 and 2016, the Brazilian Federal Police raided a child pornography ring acting inside the dark web. With these data, we build a topic-view network and compare network disruption strategies with the real police work. Only 7.4% of the forum users share relevant content, and the topological features of this core differ markedly from other clandestine networks. Approximately 60% of the core users need to be targeted to fully break the network connectivity, while the real effect of the arrests was similar to random failure. Despite this topological robustness, the overall “viewership network” was still well disrupted by the arrests, because only 10 users contributed to almost 1/3 of the total post views and 8 of these were apprehended. Moreover, the users who were arrested provided a total of 60% of the viewed content. These results indicate that for similar online systems, aiming at the users that concentrate the views may lead to more efficient police interventions than focusing on the overall connectivity.

## Introduction

In general, internet content is considered as belonging to either the surface web or the deep web depending on the possibility of regular search engines to index it. The set of web pages that for any reason are not accessible by web crawlers is typically called the deep web (*e.g*., subscription information, medical records, financial records, government resources). Due to this property, it is very hard to estimate the relative size of both layers, however, it is widely believed that the deep web is much larger than the surface web. In an even deeper layer, there is an underlying web, called the dark web, in which there is a significant effort to keep users and their data anonymous, by requiring specific configuration methods and software^1^. Precisely because of this feature, the dark web is sometimes used for illegal purposes that range from the black market to fraud services, to child pornography and terrorism. One of the most well-known platforms on the dark web is the Tor network (there are other services such as Freenet and I2P)^2^, an anonymity network originally developed by DARPA in the mid-1990s and lately made available worldwide^3^. Due to its high level of anonymity, the Tor network has been used by criminals to run illegal sites and forums, some of which have been seized by law enforcement agencies worldwide^4^.

From 2014 to 2016, Brazilian Federal Police Agents monitored the activities of individuals forming a pedophile forum on the Tor project during the so-called Operation Darknet^5^. The investigation lasted for 2 years and resulted in the identification of 182 targets (out of almost 10,000 users), several prison sentences, search warrants and in the rescue of at least 6 children that were being abused^6,7^. After that stage, the monitored message board was deactivated by a court order and the data were stored in the Federal Police servers for further analyses. Brazilian Federal Agents were then able to build a network consisting of the interaction between users mediated by the viewing of their posts. According to the National Center for Missing and Exploited Children^8^ and to the Association of Sites Advocating Child Protection’s white paper^9^, child pornography is one of the fastest-growing businesses online, with an estimated annual revenue of $3 billion USD. Child pornographic content are shared among offenders who redistribute them online, causing lifelong harm to the victims and even further trauma into adulthood. However, virtually nothing is known about the networked structure of these rings that share and view child abuse content. Even less is known about the real impact of police interventions on dark web criminal networks, mostly because of the lack of comprehensive data. These two aspects represent deep gaps in the literature of criminal networks.

The broader field of criminal networks, however, has experienced considerable growth in the last few years^10–12^. For instance, recent research studied an intelligence network gathered by the Brazilian Federal Police, showing it is highly modular and that it can be broken down with the removal of only 2% of its nodes or edges^13^. Ribeiro *et al*. have also explored the dynamical structure of political corruption networks in Brazil, showing the co-occurrence network dynamic is strictly related to the general elections^14^. Spatial and temporal regularities in crime have also been extensively explored in the context of city science and circadian rhythms^15^. In general, the data used in mathematical and physical modeling of crime derive from *a posteriori* and open access sources such as court reports, media coverage or criminal statistics. Therefore, obtaining data from official reliable sources is still today one of the key challenges in the field of criminal networks.

In order to fill the gaps discussed above, and to face one of the crucial tasks of the social physics community researching criminal networks, we study here the unique dataset of the Operation Darknet and make the whole anonymized network data available in the Supplementary Information and in the Supplementary Datasets 1 and 2. In this sense, our research sheds light on the behaviour of child-abuse rings on the dark web by showing that only 7.4% of vertices are responsible for posting the illicit media online and an even smaller number, 0.27% of individuals, concentrate half of the post views. Even though this core of criminals is tightly connected and cooperating for resources, which makes the ties hard to break, the police intervention was 60% efficient in reducing the amount of post views, out of 90% possible. Accordingly, removing the individuals that concentrate the views, instead of the most prominent topologically, could lead to a more efficient disruption. Such an approach might lead to better police interventions when tackling similar online scenarios.

## Data

The pedophile ring data were acquired directly from the dark web internet forum or message board investigated during Operation Darknet– *i.e*., an online discussion site where individuals could interact in the form of posted messages and files in conversations known as topics or threads. All user behavior was emergent, since Brazilian Federal Agents were only passively monitoring all relevant activity. When using the forum, users could interact in a variety of ways, however, the most important for us here was the interaction by topic view where a user would demonstrate their interest in another user by viewing one or several of their posts, often multiple times. In this sense, we constructed the cumulative network by aggregating the data over the entire period of observation, since we do not have access to temporal and/or spatial data. The viewing network was built accordingly— if user *i* views user *j*’s post, then a directed edge from *i* → *j* is created. If user *i* had viewed multiple topics posted from user *j*, the edge is weighted by the number of views.

## Structure

The topic network consists of a fully connected graph with 10,407 nodes and 842,247 directed edges. In directed networks each node has an in-degree *k*_in_, an out-degree *k*_out_, and a total degree *k* = *k*_in_ + *k*_out_. Each directed edge points from a source node towards a target node. The total degree variance-to-mean ratio (VMR) is *d*_all_ = 1,369.12 meaning the network degree distribution is over-dispersed. This behaviour is easily seen by the VMR for in-degree, *d*_in_ = 2,392.34 and out-degree *d*_out_ = 109.26. In fact, from the total number of nodes, 7.4% of vertices have in-degree different than zero while almost all users have non-null out-degree (10,404), meaning that only 769 individuals are responsible for posting all child pornography content, while the vast majority (82.6%) of users are only viewing the forum’s material. The resulting averages are 〈*k*_in,out_〉 = 80.93 (SD = 440.00). However, just taking the users who share content (i.e. with *k*_in_ > 0), we find an average in-degree of 1095.2 (SD = 1228.4), this is the number of unique viewers per sharer. On average each of these sharers receives 8,208.4 (SD = 25,825.6) views per post. Accordingly, most users view the posts of only a few others, while a smaller fraction of them tend to view the whole forum content.

**Figure 1:**
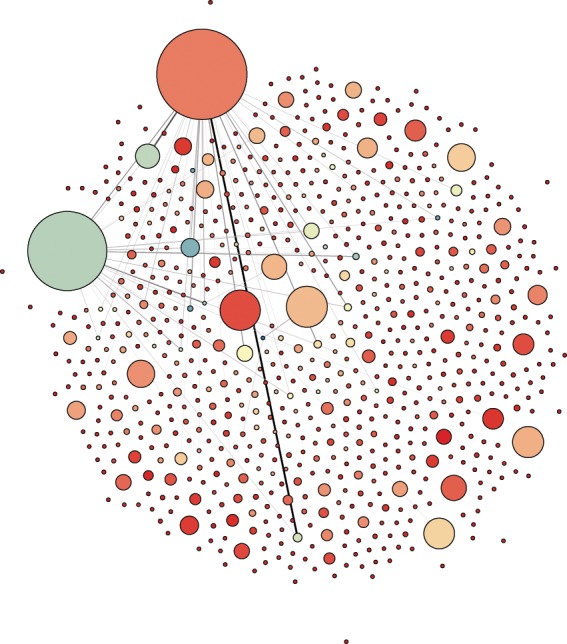
The strong component visualization with circular layout. The radii of the nodes are proportional to their in-degree, the edge weight is mapped into its darkness and the higher a node’s out-degree the closer its color is to red. (Not every edge is shown for the sake of clarity).

In directed graphs, a weakly connected component is a subgraph in which any two vertices are connected regardless of the direction of the geodesic paths. On the other hand, strongly connected components are the ones in which the subgraphs are fully connected by directed shortest paths^16^. In this sense, the studied network is weakly connected by only one single component. However, there is only one strong component comprising more than one vertex, this is of size 766 while all the other vertices are spokes that are only viewing content and not posting any relevant media (in Fig. 1 we show one possible visualization of this component). Therefore, the core of this online forum is precisely this strongly connected component which is responsible for keeping the structure functioning as a whole, while those individuals who are weakly connected don’t play a prominent structural role. Going forward, we will deal with both of these networks independently and refer to them as the “full network” and the “strong component”. For simplicity, we also label as “sharers” the users belonging to the strongly connected component and as “spokes” the ones belonging to the other components of size 1.

The topological density of a directed network is defined as the relative number of edges as compared to the possible number of connections. The density of a criminal network is usually related to the “brightness” of the system in the sense that a large number of connections among criminals means that if one actor is caught by law enforcement or intelligence agencies it would be possible, at first, to extract critical information about the local network structure and other participants from that arrested criminal^17^. On the other hand, a darker network means the direct transfer of information within the network is slowed down due to the decreased number of possible paths among criminals. Therefore, the network density informs us about the compromise between security and effective communication. In this sense, we expect clandestine networks, that operate hidden within the social fabric, such as pedophile and other illegal networks, to have very low levels of density. While the full network also has a low density *D* = 0.008, the strong component is highly dense (*D* = 0.192) in comparison with other known criminal networks^13^.This might be due to the fact that users in Tor browser forums tend to feel safe behind the secrecy of the platform and of the avatar they use. It should also be pointed out though, that with an edge representing viewing of a post, the formation of edges is more liberal than in other criminal networks which, in some cases, could require a physical encounter. In particular, the literature is scarce on networks intermediated by post viewing built in a similar fashion to the one presented here. Networks with similar mechanisms might be built using social networks or subscription services.However, at the time of this research data of this type were not available for us. Despite that, we show in Table 1 the number of nodes (N), edges (E), density (D), modularity (Q), best known topological attack (Att), generalized robustness (R) and assortativity (A) for 8 clandestine networks as well as the full and strong component of the network studied here. Even though these networks have different creating mechanisms (from intelligence to co-offending), they all share one very important underlying social feature, *i.e*., clandestinity.

**Table 1:** The table shows the following features of 10 covert networks: network size (N), the number of edges (E), the graph density (D), the average network modularity according to *Louvain* method (Q), the best known topological targeted attack, the network robustness to that attack (R, see Eq. 2) and the degree assortativity (A). BFP2013 is the Brazilian Federal Police criminal intelligence network^13^.

Network	N	E	D	Q	Att	R	A
BFP2013	9,887	19,744	4E-4	0.96	HBA	0.007	0.02
ISIS	56	30	1E-2	0.50	CI	0.020	−0.39
CAVIAR	110	3,546	3E-1	0.65	CI	0.040	−0.28
DRUG	293	337	4E-3	0.75	CI	0.040	0.06
IRA	83	184	3E-2	0.75	CI	0.060	−0.04
FIFA	450	10,859	5E-2	0.74	CI	0.060	0.24
GANGS	67	228	5E-2	0.56	CI	0.070	−0.35
9/11	61	199	5E-2	0.64	CI	0.130	−0.16
Full	10,407	842,247	8E-3	0.20	H_*i*_DA	0.055	−0.26
Strong	766	112,460	0.19	0.16	HB_*s*_A	0.347	−0.21

The ISIS dataset consists of the relationships between members of the terrorist group supplied according to the BBC^42^. CAVIAR is the dataset from an investigation of drug trafficking operating out of Canada^35^. DRUG stands for the network of drug users in Hartford, USA^43^. IRA is the network data on active Irish Republican Army members between 1970 and 1998^44^. FIFA is the network of covert elements from a recent scandal involving the Fédération Internationale de Football Association^45^. The GANGS dataset describes Italian gang members^46^. 9/11 is the network data of the bombing of the World Trade Centres in 2011 constructed from news reports^47^. The network studied in this paper is split “Full” for the whole graph and “Strong” for the strongly connected component.

Another striking feature of real networks is that they tend to show a coarse-grained structure in which groups (also called communities or modules) of densely interconnected nodes are only sparsely linked to the rest of the network^18^. This behavior is sometimes useful for criminal networks: while the tightly connected community structure makes it easier for information to spread, the rare connection between communities facilitates secrecy of operations^13^. The existence of community structures is measured by the network modularity Q, which is the difference between the fraction of edges within modules and the expected fraction if the network were random^18^, with values of *Q* close to 1 indicating highly modular structures. There are many different methods for extracting the community structures of a network. Here we used the *Leiden* method^19^, a recent modification of the *Louvain*^20^ method with improved performance (however, we obtain similar results using *Louvain* or infomap^21^). The studied strong component does not show hierarchical activities and well defined community structure, with *Q* ≈ 0.16 (the extraction algorithm identified only 3 modules with 348, 256 and 162 nodes). The average modularity for 100 Erdös-Rényi graphs with the same size and average degree is *Q* = 0.033, SD = 0.001, and for 100 configuration models is *Q* = 0.042, SD = 0.001. Typical values of *Q* for real-world modular networks range from 0.3 to 0.7^18^, which indicates that the network studied here (with *Q* ≈ 0.16) is not strongly modular. This feature differentiates it from other known criminal networks (see Table 1). The full network yields a similar value (*Q* ≈ 0.20) with the major cluster identified being the strong component.

## Degree Distribution

As mentioned above, the degree distributions are over-dispersed. The in-degree distribution demonstrates this with a small number of vertices’ in-degrees being far above the mean. As the in-degree of a vertex corresponds to the number of different users viewing that node’s post, these are distributors whose content is highly visible. The out-degree distribution is capped by the number of users sharing content, we observe that one vertex views content from 602 of the 769 providers.

**Figure 2:**
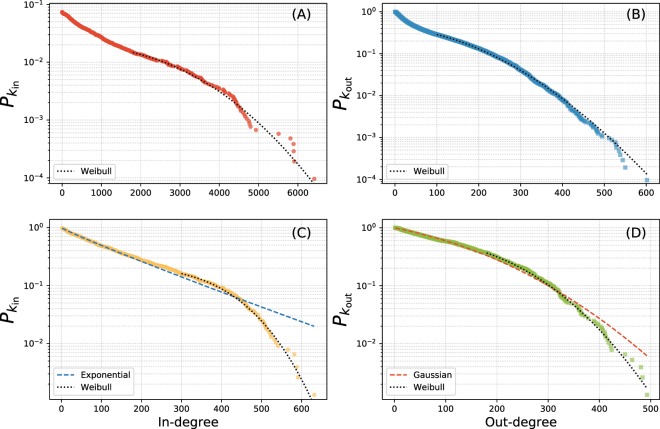
The in and out-degree distributions for the full network and the strong component. Upper panels (A) and (B): the complementary cumulative degree distributions for the full network *P*_*k*_ for the in-degree in panel (A) and the out-degree panel (B). The tail of each are well fitted by Weibull distributions. Lower panels (C) and (D): the degree distribution for the strong component with the in-degree distribution displayed in panel (C), this receives the best support from an exponential distribution (dashed line), however, fitting to the tail a Weibull (dotted) is best supported. In panel (D) the out-degree distribution is shown, the whole distribution (dashed line) is well fitted by a normal distribution (dashed) and the tail, again, by a Weibull (dotted).

The heterogeneity of the degree distribution is a key feature in studying network robustness^22–24^. For instance, networks with random degree distributions fall apart after the failure of a critical number of nodes. On the other hand, networks with heavy-tailed degree distributions are usually very fragile to targeted attacks to central nodes and full fragmentation is attained after a small fraction of vertices is removed from the system^16^. Most known criminal networks have heavy-tailed degree distributions^13^. Using maximum likelihood estimators, the following distributions are tested to the full and tail of the distribution: power law, exponential, Weibull, normal (Gaussian), log-normal and Poisson. The Akaike Information Criterion for small sample sizes is used to select the appropriate model from the log-likelihoods^25^. In each case the complementary cumulative distribution function is displayed as this has less noise, however, the fits are made to the original degree data. The degree distributions for the full network are shown in the upper panels of Fig. 2 with both the tails being well fitted by Weibull distributions (although a log-normal cannot be ruled out for the in-degree distribution). The lower panels of Fig. 2 shows the in-degree and out-degree distributions only for the strong component of the network. The entire distributions are well-fitted by an exponential and normal (Gaussian) respectively (dashed lines). Fitting to just the tail of the distribution, Weibull distributions are preferred again (dotted lines), however, log-normals cannot be ruled out in either case. In each case, we see a small number of users both viewing content and having their content viewed disproportionately compared to the rest.

**Figure 3:**
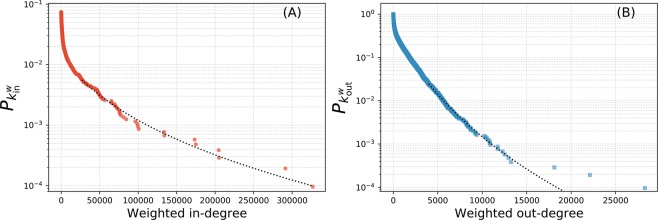
The weighted degree (*k*^*w*^) distribution for the full network. The in-degree distribution is shown in panel (A) and the out-degree distribution is displayed in panel (B), both with fitted log-normal distributions.

Figure 3 shows the weighted degree-distribution for the full network. The full distribution for the weighted in-degree is best fitted by a log-normal and the out-degree by a Weibull once again (not shown). Fitting to just tails, the best support is received from a log-normal distribution in each case (dotted lines). However, each distribution received some support here and therefore cannot be ruled out. Note that here, the weighted in-degree of a vertex represent the number of times all of that user’s posts were viewed. The top ten weighted in-degrees are all over 100,000.

## Rich-club Effect

**Figure 4:**
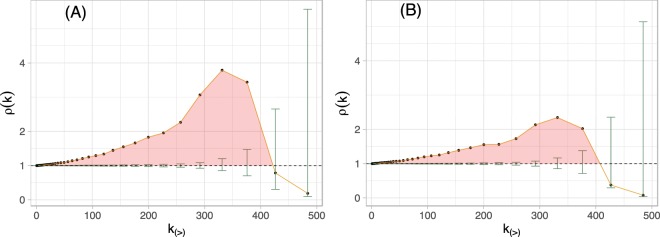
The rich-club coefficient *ρ* for the strong component as a function of the total degree *k* with the respective 95% confidence intervals. Weight reshuffle is shown in panel (A) and edge reshuffle in panel (B).

In networks from a variety of fields, the most prominent individuals tend to connect among themselves more commonly than at random, building a sort of “exclusive” club as a means of keeping control over the networked system^26–29^. This tendency, which is called the rich-club effect, is poorly explored in criminal networks mainly because of the lack of comprehensive data. However, this feature is very relevant in criminal networks’ robustness to police intervention since a high rich-club coefficient might result in tightly connected hubs making the network robust to hub removal. The topological rich-club coefficient is defined for directed graphs as the ratio between the number of edges in the network and the possible amount of edges for nodes with total degree greater than *k* = *k*_*in*_ + *k*_*out*_, for every total degree *k*, that is:

1 $$\phi (k)=\frac{{E}_{\ge k}}{{N}_{\ge k}({N}_{\ge k}-1)},$$

where *N*_≥*k*_ is the number of vertices with degree higher than or equal to *k*, and *E*_≥*k*_ is the number of edges among these nodes^30^. To get statistically significant results, *ϕ*(*k*) should be rescaled by the average *ϕ*_null_(*k*) expected for a random graph with same degree distribution; we do so by generating 1,000 ensemble of random networks using both link and weight reshuffle^31^. Therefore, if *ρ*(*k*) = *ϕ*(*k*)/*ϕ*_null_(*k*) > 1 the network displays a positive rich-club effect. In this sense, the shaded area in Fig. 4 highlights the regions where the rich-club coefficient is statistically significant, with a 95% confidence level. As depicted in Fig. 4, the strong component shows a rich-club coefficient that becomes more prominent as the connectivity increases, reaching a peak at *k* > 325 and then vanishing for *k* > 410. This means that in fact there is a high-degree elite of individuals that tend to associate with one another feeding the online forum with pedophilic media posts and reciprocal viewing. In such a scenario, the presence of a significant positive rich-club is usually related to cooperation between individuals in the upper elite to concentrate post views. The opposing case of a statistically significant anti-rich-club is sometimes associated with competition between individuals for the network resources, post views in this case. However, the top *k* > 26% sharers do not show significant rich-clubs, and one cannot categorically say if there is competition or cooperation taking place for these individuals.

The rich-club effect is actually a specific definition from a more generic measure of degree correlations called assortativity or homophily. While the assortativity essentially measures how connected nodes with similar degree are, the rich-club is only concerned with the connectivity of nodes with degree above a certain threshold. The two measures are not in conflict. For instance, a network consisting of only hubs and spokes, with hubs well connected among them, is typically disassortative (spokes connected to hubs) with the presence of a strong rich-club effect (hubs connected among themselves). This is precisely the virus-like topology of the network studied here. In social and business networks, for example, highly popular individuals tend to connect to others with similar popularity to maintain reputation and social status. However, some online networks are known to be disassortative^32^. In the particular case studied here, it appears that users are not really interested in keeping an online social status or reputation, in a way there is no strong competition among users. Presumably, individuals are mostly interested in filling their need for child pornography regardless of what source it comes from. Such behaviour would be consistent with the disassortative mixing of −0.21 of the strongly connected component and −0.26 of the full network.

## Dismantling Approaches and Police Intervention

Law enforcement resources are limited and investigations usually face multiple practical and legal limitations. Therefore, it is crucial to know the minimum set of nodes that if removed would result in the largest breakdown of a criminal network - a problem that, in abstract, is usually known in network science as the minimum dismantling set, a NP-hard problem that has currently no solution for large-scale networks^33,34^. From the network science point of view, a criminal network is a graph consisting of criminals (vertices) connected by relationships (edges) that might be of many natures (telephone calls, co-offending, intelligence-based, etc)^35^. In general, law enforcement interventions aim to identify and arrest criminal actors. This procedure has a simple topological interpretation: when a criminal is put in jail, a fraction of his/her relationships is cut^12^. This course of action is known to be less effective than neutralization strategies such as the total removal of edges (solitary confinement) or the removal of nodes (full resocialization or death)^13^. However, when online networks are considered, nodes are only avatars of real people and simply arresting a criminal results in the removal of the online user from the network – a typical neutralization strategy.

Most network disruption strategies are divided into two approaches: non-adaptive (or simultaneous) attacks and adaptive (or sequential) attacks. In the non-adaptive approach, the list of targets is computed only once, before the disruption process begins. In the adaptive approach, the list of targets is updated after each deletion by the recalculation of the centrality index used to sort the nodes. Consequently, adaptive attacks demand more processing time, but on the other hand, they usually produce more damage since they account for the changes in the network topology due to the removal of nodes and edges^36^. Over the years, several heuristic attack strategies have been proposed to break down complex networks with the least number of removed nodes^37–40^. However, the network dismantling problem is an open research area and no ultimate attack strategy is known so far. The order parameter *G*(*q*) = *N*_*q*_^*s*^/*N*^*s*^ is usually defined to capture the response of a network to node and/or edge removal, where *N*_*q*_^*s*^ is the size of the largest strongly connected component after a fraction *q* of nodes is removed, and *N*^*s*^ is the size of the original network’s largest strongly connected component. It actually measures the relative size of the largest strongly connected component of the network as a function of the fraction of nodes deleted. In this sense, the generalized robustness of a network to a given disruption strategy^36^ usually considers the relative size of the largest remaining connected component of the network during the attack procedure as:

2 $$R=\frac{1}{{N}^{s}(1-{G}_{min})}\mathop{\sum }\limits_{q=0}^{{q}_{max}}G(q),$$

where *q*_*max*_ is the point at which the attack ends and *G*_*min*_ is the value of the relative size of the largest strongly connected component at *q*_*max*_.

**Figure 5:**
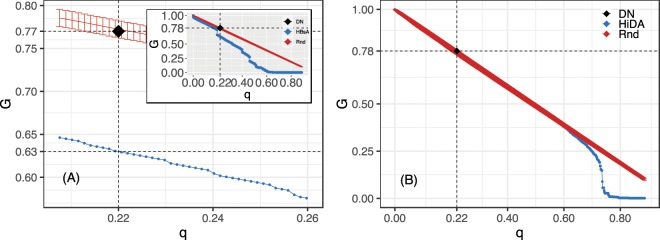
The figure shows the fragmentation process. In the left panel (A), the inset figure shows the size of the largest strongly connected component as a function of the fraction of nodes removed according to the best topological attack (HB_*s*_A - blue curve), the mean and error bars for a 1,000 random attacks (red curve, Rnd), and the real police intervention result (black diamond shape, DN). In this case the network only breaks completely after 60% of removals. The main figure shows a zoom in near the real police intervention which resulted in a 23% decrease of the strongly connected component, while the topological attack resulted in a 37% breakdown (1.6 times more efficient). The right panel (B) shows the same process but now considering the relative size of the largest connected component (regardless of strong and weak components). Note that in this case even the best topological attack (high in-degree adaptive attack, H_*i*_DA) in the region that real police intervention took place is as good as random and the network only breaks after almost 80% of disruption.

In many real and artificial networks, high-betweenness adaptive (HBA), high-degree adaptive (HDA), collective influence (CI), and module-based (MBA) attacks tend to decrease the largest connected component of complex graphs very efficiently^41^. The HBA algorithm consists of sorting nodes according to the betweenness centrality, removing the highest-ranked one, recomputing the index and so forth. The HDA procedure simply substitutes the betweenness centrality by the degree (either total, in or out). The collective influence, which is known to be close to the minimum dismantling set as long as the network has local tree-like structure, consists of the same procedure as before but with the collective influence centrality defined for node *i* as $C{I}_{\ell }(i)=({k}_{i}-1)\,{\sum }_{j\in \partial B(i,\ell )}({k}_{j}-1)$, where *k*_*j*_ is the degree of the nodes in the edge of a ball of radius *l* around node *i*, and *k*_*i*_ is the degree of node *i*^39^. The sum of weights of the adjacent edges of a given node is called the node’s strength *s*. For HBA the shortest path algorithm can be generalized to take *s* into account (call it HB_*w*_A), and the same goes with HDA (call it HSA). Figure 5 shows the size of the largest strongly connected component (left panel) as a function of the fraction of nodes removed and the size of the largest connected component regardless of strong and weak components (right panel).

The full network is fragile to the removal of a small subset (≈7%) of the vertices. However, these are all in the strong component. Despite the in-degree distributions showing a small number of users sharing most of the content, the strong component is very dense. As shown in Fig. 2, the degree distributions for the strong component are well fitted by exponentials and Gaussians. This also indicates that the strongly connected core should be very resilient both to targeted attacks and random failures. In fact, it would take the random removal of all users for the network to be fully disconnected and the removal of almost 60% of individuals according to the best-targeted attack strategy (see Fig. 5).

We now assess the outcome of the police investigation. To do that, we first explain *en passant* how police targeted criminals during the Operation Darknet. Firstly, investigators defined a qualitative pyramid of priorities according to the seriousness of their conduct under the Brazilian Penal Code: the most sensitive users were the ones that were apparently committing child abuse in real life, followed by heavy content sharers and then by users that only downloaded material. Afterwards, users started having their real life identify uncovered by lifting the secrecy of the Tor browser cloaking mechanism for each of them– details of such are reserved. After that, the traditional criminal investigation took place, and investigators searched for further evidence of the involvement of each individual in related crimes. In fact, each criminal case demonstrated its own idiosyncrasies and investigative challenges. Besides, some caveats obliged investigators to bypass the seriousness pyramid a few times. For instance, even though the police’s aim was to arrest the largest number of criminals, the technical process of identifying real individuals out of the Tor browser was time consuming and dependent on many practical issues such as the level of expertise on cloaking of each user, while due to legal requirements the investigation had a limiting time frame. The aftermath of the investigation was 176 criminals identified in real life and arrested for either sharing or storing child pornography media and 6 for child abuse— out of this total, 170 were sharers (22% of the strong component) and 12 were spokes.

The main panel of Fig. 5(A) is a zoom in the fragmentation curve that shows the topological effect on the strongly connected component of removing precisely those individuals that were arrested by the police, 22% of sharers and a 23% damage to the strong component. This result lies inside the error bars of the random removal strategy and damaged 1.6 times less the core when compared to the best topological attack. The removal of the 12 spokes does not significantly affect the network. Accordingly, these data indicate that the police investigation was highly effective in identifying individuals belonging to the network core, *i.e*., to the strongly connected component, since 93% of the identified users are sharers, and led to the arrest of a significant amount of criminals, 22% of the strong component. However, the sharers arrested by the police were not as structurally important as the ones identified by the HB_*s*_A method (by a factor of 1.6). On the other hand, the network topology is such that even the best topological intervention could only fully atomize the system after a large removal of more than 60% of all sharers. Moreover, if we consider the only the largest connected component, regardless of edge direction, the network is even more robust, only fragmenting after almost 80% of removals, while topological attacks and police interventions are as good as random failures.

**Figure 6:**
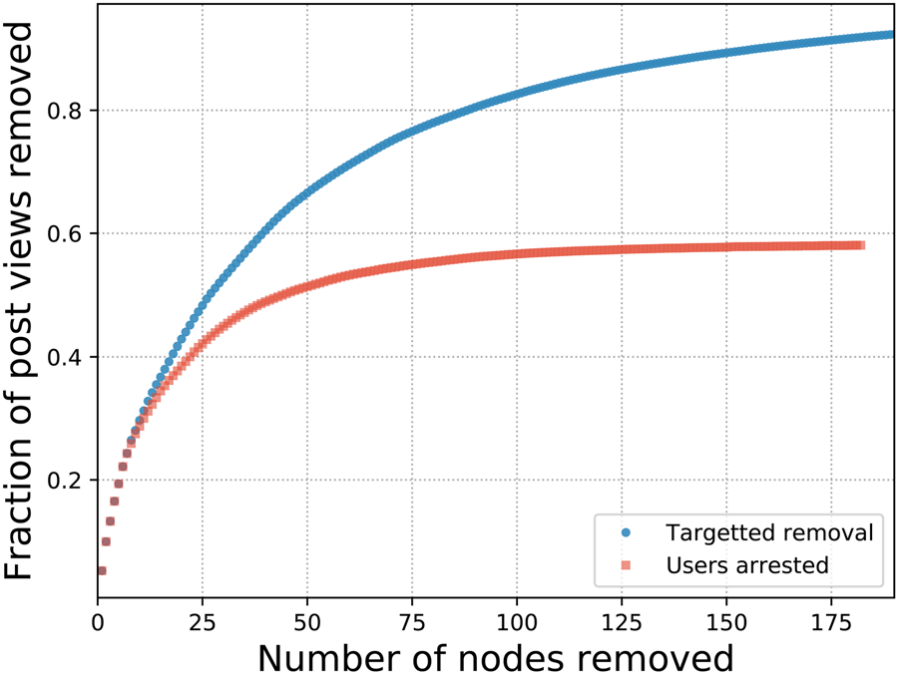
The fraction of post-views removed by targeting users of highest weighted in-degree (blue circles) and the actual arrests (red squares). Initially the arrests are very successful, quickly disrupting 40% of the post-views.

Although the topology of the network is hard to break down, we observe from the weighted in-degree distribution that the majority of posts are shared by a small fraction of the users. In Fig. 6 we show the result of removing nodes in order of weighted in-degree (blue circles). Removing the top 100 nodes reduces the post views by 82.6%. The red squares represent the removal of the arrested users. Initially this is highly successful at disrupting the posts with 8 of the top 10 sharers arrested. With 20 arrests 38.5% of a possible 42.8% reduction of posts is achieved. Subsequent targets are less optimally selected, so that the total arrests reduced the posts by 58.1% of a possible 91.9%. In this sense, removing the top 10 vertices according to the number of post views reduces the total number of views by 29.2%. Removing the top 28 vertices reduces this by 50.2%. Therefore half of the post views in the whole network can be attributed to only 28 users posting content.

## Discussion

In this research, we have presented (we believe for the first time) a criminal network inter-mediated by topic view in which pedophiles interacted in a dark web forum using Tor browser. The original directed and weighted network consists of 10,407 users, out of which 9,641 are weakly connected to a central core of only 766 strongly connected individuals. The peripheral 9,638 individuals didn’t post any content online, being what we call spokes. On the other hand, the 766 individuals belonging to the strongly connected core were actively sharing and viewing content, being the ones actually responsible for structuring the whole criminal enterprise. The weakly connected users might be composed of individuals that joined the forum out of curiosity or paedophilic inclinations. However, this structure could be seen as a gateway to more serious criminal activity happening in the network core, a hypothesis that should be addressed in future research when dynamic data are available. As we pointed out, the weakly connected periphery of users is composed only of leaves which have no particular structural role, therefore the main focus is on the strongly connected component.

Even though viewing networks are virtually nonexistent in the literature, we were able to compare the child abusers network with other networks that share the common social feature of clandestinity. The core of strongly connected criminals has many stark differences to other known criminal networks. One of these disparities is that the core has no well defined modular structure, while typical criminal networks tend to display highly coarse-grained topologies. This feature has two relevant interpretations. First, this lack of modular structure points to users not having interests in particular topics of the forum. The second interpretation is related to the fact that recent evidence suggests that modularity arises in competitive scenarios as organized crime, financial markets or predator-prey biological systems. However, the core of the network under study here consists of individuals cooperating to meet their pathological needs. Moreover, clandestine networks’ strategies to cloak their activities from law enforcement investigations is usually reflected by low graph densities. Nonetheless, the strong component of this network is highly dense. In fact, users of the dark web, more precisely of the Tor browser, feel safely hidden by avatars and by several levels of anonymity. Therefore, perhaps users tend to act freely on the online forum, without worrying about cloaking their activities or the local network structure. Besides, the anonymity of a fantasy avatar hinders sociological mechanisms such as the search for indirect prestige through appearances, as shown by the network core’s degree disassortativity. Indeed, users connect among themselves by post views if that particular content indulges their taste for child abuse media, regardless of who produced it. However, it should be pointed out that in a viewing network edges are created much more easily than in some other criminal networks, some of which require a physical interaction. This might also contribute to the high value of the density. Even though the network is highly disassortative, there is a peak of the rich-club coefficient which vanishes for highly connected individuals. The lack of rich-club effect for the highest active criminals indicates the network might be robust to degree-based removals. In fact, the strong network only breaks after the removal of ~60% of the users according to HB_*s*_A (or even after ~80% according to HDA if link direction is not taken into account). This is again a stark contrast to most criminal networks which seem to be much more fragile to targeted attacks. This robustness feature is directly related to the degree distribution of the network. While organized crime networks often have heavy-tailed degree distributions, this one is much more homogeneous with a light-tail distribution.

The police force was very effective in identifying criminals belonging to the core— out of 182 users arrested in real life, 170 (93%) belonged to the core while only 12 were weakly connected spokes. However, the targeted attack was only as effective as random failures; even the best topological attack could have been only 1.6 times more effective. Accordingly, the best approach seems to be maintaining sting operations until a large amount of the core is disrupted. Another possibility would be to monitor different online forums searching for recidivist users that couldn’t be arrested due to time and/or legal limitations of previous investigations. However, aiming at the users that attract the most views should decrease the amounts of viewing much easier than breaking the whole structure. Despite the network being very robust, we can still significantly disrupt the amount of views. Only 10 users contribute to almost one third of the post views and a further 16 bring this to half. Eight of those top 10 users were arrested in the investigation, *i.e*., the police had 80% of accuracy in arresting users that significantly attracted the majority of views. Therefore, even while we can’t easily disrupt the entire network in a traditional sense, we can decrease the content provided without using structural network analysis. In this sense, the real investigation resulted in 60% of post views removed with the arrest of 182 users, a highly effective achievement by the police. However, the investigation could have been targeted to a few more users from these top distributors to achieve a higher disruption of ~90%. By removing individuals that concentrate the views, the remaining activity, even though structurally cohesive, would reduce to only a small amount of views, hampering the main activity of the forum and indirectly disrupting the system.

Future research may aim to gather data from other dark web’s forums to help build a multiplex network able to identify deeper patterns of criminal activities that are not usually targeted by police investigations, but that frequently hop between forums (layers) and thence keep the overall child abuse activity working on the dark web. Another possible future research might be getting access to viewing data through cooperation with social network services in order to compare these results to other networks (licit and illicit) with similar building mechanisms.

We hope these results help investigators worldwide to better tackle this horrific phenomenon and that this research can be useful in devising effective strategies to break down these criminal rings.

### Ethics statement

The original data were handled only by Brazilian Federal Police Officers with legal clearance to do so, and under the supervision of a Federal Judge and Prosecutors. All data handling was in accordance with the Brazilian law for data protection (Act No. 13,709 from 2,018), the Brazilian individual rights act (Brazilian Constitution from 1,988), the Brazilian Criminal Code (Act No. 2,848 from 1,940), the Brazilian Criminal Procedure Code (Act No. 3,689 from 1,941), and the Brazilian Federal Police internal procedures. All data were analysed only after a complete cryptographic anonymization procedure.

## Supplementary information

Information.

Dataset 1.

Dataset 2.

## Data Availability

The network data studied in this paper are available in the Supplementary Datasets 1 and 2 along with a Supplementary Information file explaining it.
