# Supplementary material for: Assessing police topological efficiency in a major sting operation on the dark web
Source: Sci Rep. 2020 Jan 9;10:73. doi: 10.1038/s41598-019-56704-4 (PMC6952354; doi:10.1038/s41598-019-56704-4)
Supplement: Supplementary file 1 — Information. [file 41598_2019_56704_MOESM1_ESM.pdf]

Assessing police topological efficiency in a major sting operation on the dark web

Bruno Requião da Cunha[1,2,*], Pádraig MacCarron[2,3], Jean Fernando Passold[1], Luiz Walmocyr dos Santos Júnior[1], Kleber A. Oliveira[2], and James P. Gleeson[2]

[1]Rio Grande do Sul Superintendency, Federal Police, Porto Alegre, 90160-093, Brazil

[2]MACSI, Department of Mathematics and Statistics, University of Limerick, Limerick, V94 T9PX, Ireland

[3]Centre for Social Issues Research, University of Limerick, Limerick, V94 T9PX, Ireland

[*]cunha.brc@dpf.gov.br

This is the README file for dataset of the post-viewing aggregated network acquired by the Brazilian Federal Police during the Operation Darknet phases I (2014) and II (2016), which investigated an illicit forum of child pornography sharers and consumers on the Deep Web's Tor browser. In this network, nodes are the forum's users and a direct edge from "i" to "j" means user "i" viewed a thread posted by "j", the weight representing the number of times "i" visualised any content posted by "j". The edge list (Supplementary Dataset 1) is a 842,248 x 3 matrix, the first column is the "from" node, the second the "to" node, and the third is the edge's weight. The Supplementary Dataset 2 is a list containing the labels for each of the users that were actually targeted by Brazilian Federal Police Agents as the aftermath of the investigation.
